# Supplementary material for: Flow Cytometric Analysis and Sorting of Murine Enteric Nervous System Cells: An Optimized Protocol
Source: Int J Mol Sci. 2025 May 18;26(10):4824. doi: 10.3390/ijms26104824 (PMC12112686; doi:10.3390/ijms26104824)
Supplement: Supplementary file 1 [file ijms-26-04824-s001.zip › ijms-3544777-supplementary.pdf]

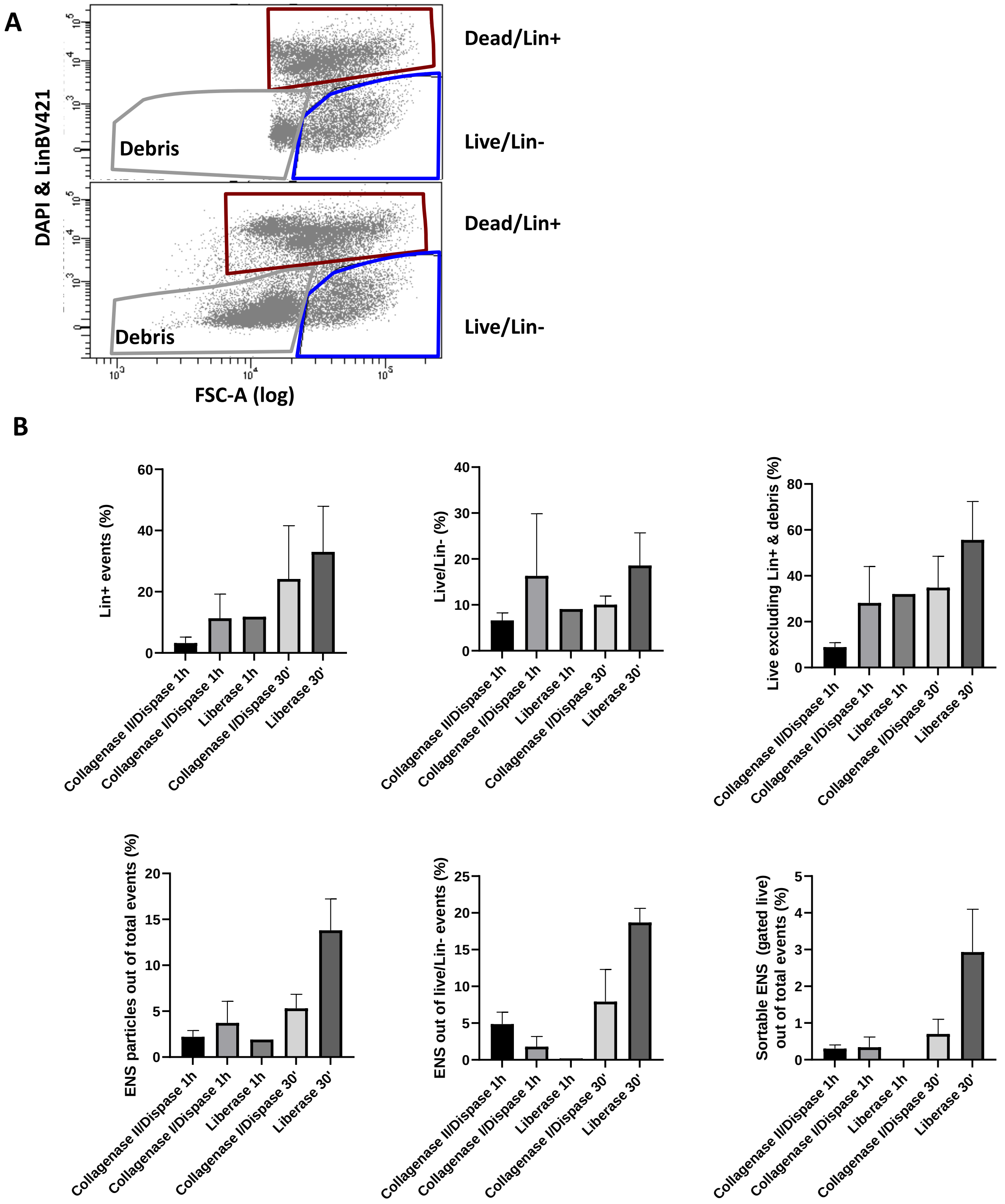

Figure S1. Extended results relative to Figure 2 and 3: (**a**) Example of how the threshold on FSC was set to include all the smallest DAPI$^+$ events. Log scale is used here to better visualize the low FSC region. Upper plot: due to high threshold the lowest DAPI$^+$ events are cut. Lower plot: reducing the threshold all the DAPI$^+$ events are included, leaving a safety margin. However, also more debris is included; (**b**) Extended quantification of the samples obtained with different protocols. Liberase at 1h is also included (N=1), which was discontinued based on the evidence that these samples were visibly over-digested. All the other samples are expressed as mean ±SD (N=3). **Upper left**: percentage of Lin$^+$ events. Due to the RBC lysis step, Lin$^+$ events are fewer with the human protocol. However, this does not significantly improve the raw percentage of live/Lin- cells (**upper middle**) or ENS. The percentage of Lin$^+$ cells is variable and tends to be higher with the 30' protocols. Due to the interference of Lin+ cells and debris, the evaluation of cell viability produces high variability, which is more evident with the 1h (over-digested) Collagenase I/Dispase sample. **Upper right**: net viability comparison excluding Lin$^+$ events and debris. This allows for a definitely cleaner comparison of cell viability and reduced standard deviations. **Bottom left**: unbiased comparison of the raw percentage of all CD56^{high}/ENS particles showing that 30' Liberase outperforms all the other protocols. **Bottom middle**: fraction of ENS events relative to the live/Lin$^-$ region. **Bottom right**: fraction of ENS events in the live/Lin$^-$ region relative to all events (i.e. the sortable fraction out of the total). The last two graphs indicate that 60'Liberase has zero ENS events in the live region (no ENS cells to be sorted), confirming that these samples are over-digested.

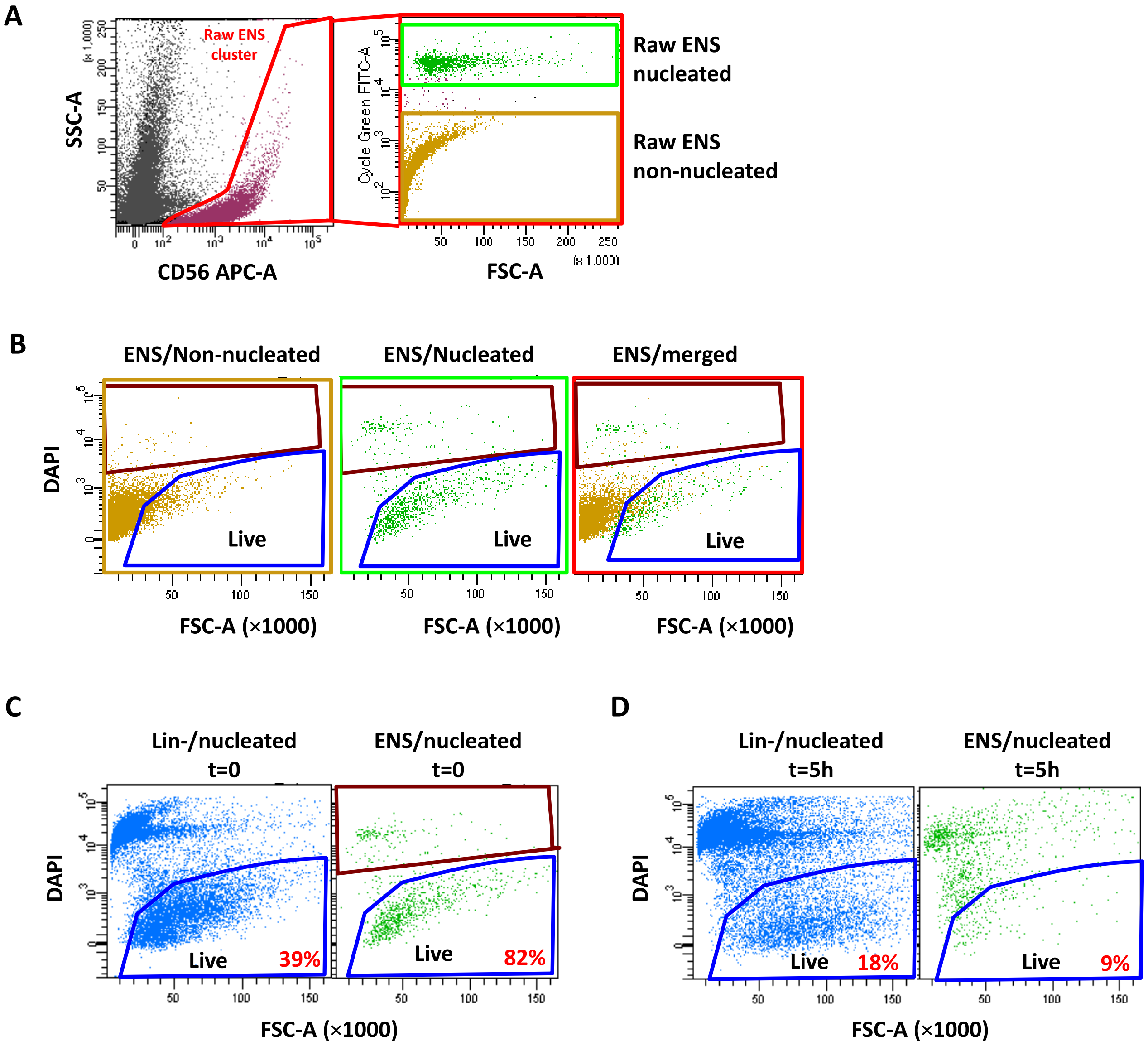

Figure S2. Extended results relative to Figure 2 to 4: (**a**) From the raw ENS events selected as in Figure 2a (left plot) live nucleated cells are divided from non-nucleated debris by means of Dye Cycle Green staining (right plot); (**b**) Nucleated and non-nucleated raw ENS events are plotted in a FSC-A vs DAPI plot to show the distribution of live cells, dead cells, and debris. Notably, these regions well match the gates on live vs. dead and debris applied in Figure 1 and Figure 2, offering a good validation of that strategy; (**c**-**d**) Examples showing that ENS cells are quite sensitive and dye faster than other cells in the same sample: (**c**) Analysis of all Lin- cells (left) and ENS cells (right) immediately after dissociation and staining; (**d**) samples analyzed after 5 hours on ice. Reducing all the steps that lead to longer processing and storage appears essential for optimal ENS-cell viability and recovery. This staining and gating strategy can be used to evaluate the quality of samples with respect to ENS cells or any other population under analysis, driving a correct selection and application of alternative dissociation protocols

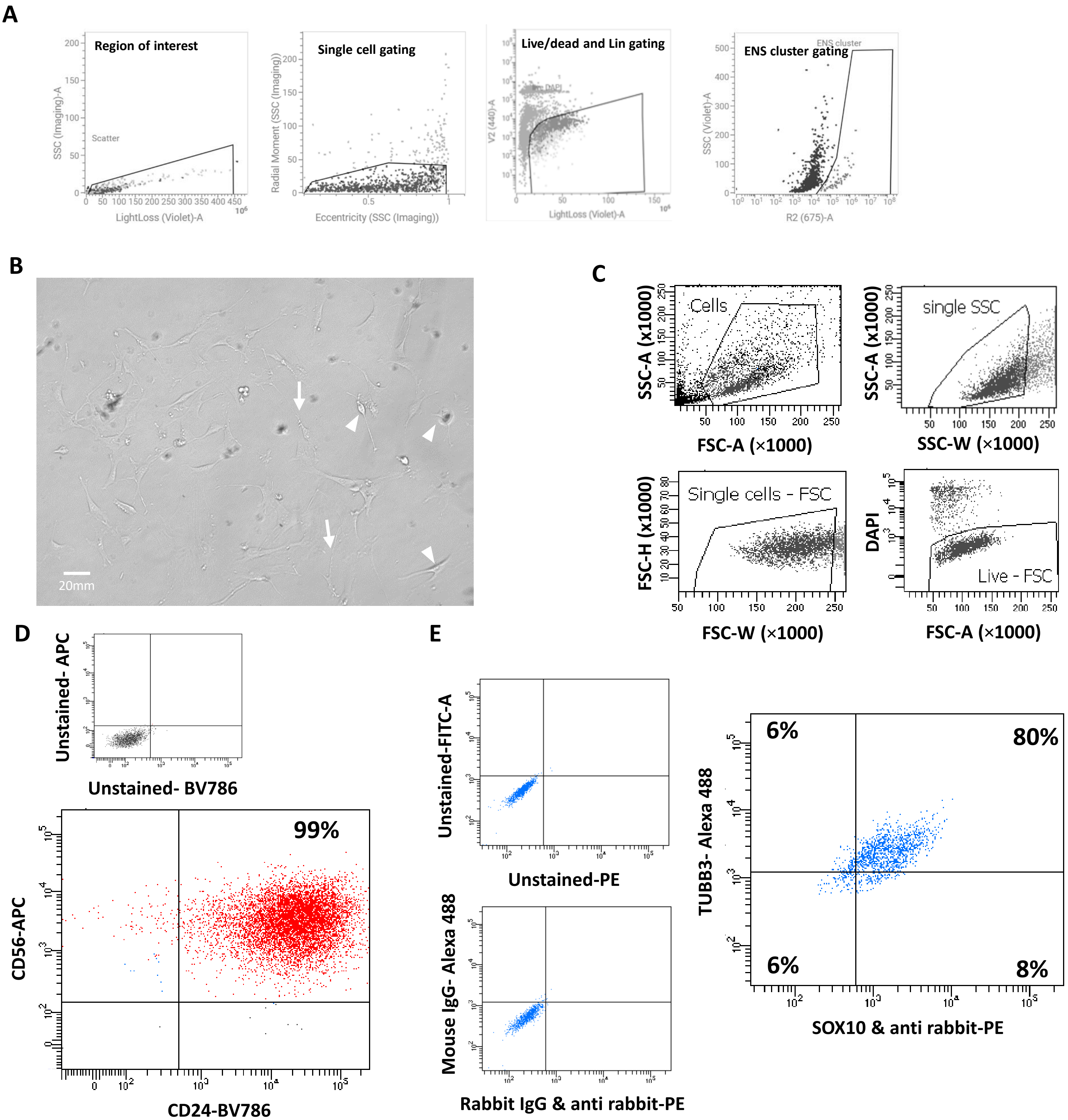

Figure S3. FACS analysis of mouse ENS cells sorted by FACS Discover and cultured *in vitro*: (**a**) Synthesis of the gating strategy used for sorting on FACS Discover. A threshold was applied and the region was selected using the parameter "light loss" on the violet laser (corresponding to FSC) vs SSC on the imaging channel (blue laser). Single cell gating was applied using the imaging parameters (blue laser) "eccentricity" vs "radial moment". Although DAPI staining for live/dead and Lin-BV421 staining could theoretically be separated using spectral unmixing, they were visualized together on the second channel of the violet laser (V2), following the same simplified strategy previously used with the FACS Aria. The ENS cluster was subsequently identified using CD56-APC on the second channel of the 633 nm laser (R2). Given the simplicity of the staining panel and the absence of spillover between distant channels, spectral unmixing was not applied, and raw channel data were used; (**b**) Transmitted light image of sorted mouse ENS cells at passage 1, captured using a 10x magnification. Examples of cells with features consistent with neuronal and glial cells are indicated respectively, with arrows and arrowheads; (**c**) Analysis of *in vitro* cultured ENS cells using FACS Aria. Preliminary gates for the selection of the region of interest containing cells, followed by single cell gating on forward and side scattering and live gating on DAPI; (**d**) ENS cultured cells are positive for both CD56 and CD24. Unstained control sample (top plot) and cells stained for CD56 and CD24 (bottom plot); (**e**) ENS identity was confirmed by TUBB3 and SOX10 staining on live-sorted and formalin fixed cells. Plotted vs unstained and control antibodies, ENS cells were almost entirely positive for both markers , confirming the pattern observed with the original ENS cluster.
